# Thrombospondins: A Role in Cardiovascular Disease

**DOI:** 10.3390/ijms18071540

**Published:** 2017-07-17

**Authors:** Dimitry A. Chistiakov, Alexandra A. Melnichenko, Veronika A. Myasoedova, Andrey V. Grechko, Alexander N. Orekhov

**Affiliations:** 1 Department of Fundamental and Applied Neurobiology, Serbsky Federal Medical Research Center of Psychiatry and Narcology, 119991 Moscow, Russia; 2 Laboratory of Angiopathology, Institute of General Pathology and Pathophysiology, Russian Academy of Medical Sciences, 125315 Moscow, Russia; zavod@ifarm.ru (A.A.M.); myika@yandex.ru (V.A.M.); 3 Federal Research and Clinical Center of Intensive Care Medicine and Rehabilitology, 109240 Moscow, Russia; avg-2007@yandex.ru; 4 Institute for Atherosclerosis Research, Skolkovo Innovative Center, Moscow 121609, Russia; a.h.opexob@gmail.com

**Keywords:** thrombospondins, cardiac remodeling, cardiac hypertrophy, heart failure, atherosclerosis, myocardial infarction, cardiac fibrosis

## Abstract

Thrombospondins (TSPs) represent extracellular matrix (ECM) proteins belonging to the TSP family that comprises five members. All TSPs have a complex multidomain structure that permits the interaction with various partners including other ECM proteins, cytokines, receptors, growth factors, etc. Among TSPs, TSP1, TSP2, and TSP4 are the most studied and functionally tested. TSP1 possesses anti-angiogenic activity and is able to activate transforming growth factor (TGF)-β, a potent profibrotic and anti-inflammatory factor. Both TSP2 and TSP4 are implicated in the control of ECM composition in hypertrophic hearts. TSP1, TSP2, and TSP4 also influence cardiac remodeling by affecting collagen production, activity of matrix metalloproteinases and TGF-β signaling, myofibroblast differentiation, cardiomyocyte apoptosis, and stretch-mediated enhancement of myocardial contraction. The development and evaluation of TSP-deficient animal models provided an option to assess the contribution of TSPs to cardiovascular pathology such as (myocardial infarction) MI, cardiac hypertrophy, heart failure, atherosclerosis, and aortic valve stenosis. Targeting of TSPs has a significant therapeutic value for treatment of cardiovascular disease. The activation of cardiac TSP signaling in stress and pressure overload may be therefore beneficial.

## 1. Introduction

The function of the extracellular matrix (ECM) is not only limited by providing structural support and immobilization of cells. Functional significance of the ECM also relies in mediating cell-cell and cell-matrix contacts, signaling conduction, and triggering cell adhesion, motility, and differentiation. The composition and functional properties of the ECM vary depending on the cell type and tissue/organ specificity. For example, in the cardiovascular system, the ECM is involved in maintaining structural continuity of the heart and blood vessels, providing an essential scaffold for cell attachment and functioning, control of cell growth, viability and death, regulation of diastolic stiffness, and performing tissue repair/remodeling in a case of cardiovascular damage and inflammation [1]. In the ECM, structural changes induced by local microenvironment can lead to functional matrix alterations. Changes in the matrix may be then conducted to adjacent cells and affect their activity and behavior. For example, the cardiovascular ECM mediates blood flow-induced mechanotransduction [2,3] and adaptive responses of vascular cells and cardiomyocytes to various stress stimuli [4,5].

In cardiovascular pathology such as atherosclerosis, arterial restenosis or heart failure, ECM-associated responses are frequently maladaptive and may lead to adverse tissue remodeling and fibrosis [6,7]. Inflammatory, profibrotic, prooxidant, hypoxic, and other pathological stimuli may induce substantial modifications and impair matrix turnover, which in turn may cause qualitative and quantitative changes in matrix architecture and composition by increasing content of certain ECM proteins and decreasing amount of other matrix components [8,9]. For instance, in failing hearts of patients with dilated cardiomyopathy, significant changes in the content of some non-fibrillar matrix and matricellular proteins were found. Implementation of the mechanical unloading of the left ventricle by left ventricular assist device resulted in the restoration of the fibrillar ECM and basement membrane and improved clinical outcome [9].

Matricellular proteins comprise non-structural ECM proteins that modulate cell function and behavior. Matricellular proteins include thrombospondins (TSPs), tenascins, periostin, osteopontin, CCN proteins, and osteonectin/secreted protein acidic and rich in cysteine (SPARC) [10]. Tenascins contain three members (tenascin C, tenascin R, and tenascin X) that are especially abundant in developing embryonic tissues like cartilage, tendon, bone, and nervous system where these proteins promote migration, proliferation, and differentiation of stem and lineage-specific progenitor cells [11,12]. Periostin plays multiple physiological and pathogenic roles including regulation of mesenchymal differentiation in the developing heart, tissue repair, and involvement in cancer and valvular heart disease [13,14,15]. Osteopontin triggers biomineralization, bone remodeling, and immunity as well as pathological ectopic calcification [16,17,18]. Osteonectin is a Ca^2+^-binding protein whose primary function is to contribute to osteogenesis by conducting biomineralization of the bone and cartilage [19]. CCN proteins include at least six members (CCN1–6) with a complex multidomain structure that provides an option to bind numerous ligands and participate in a variety of biological processes such as angiogenesis, inflammation, tissue repair, fibrosis, and carcinogenesis [20]. Like CNN proteins, TSPs have several functional domains and indeed are able to interact with multiple partners. TSPs are abundantly distributed in various tissues and organs including the cardiovascular system. At steady state, TSP expression is low but can be up-regulated in response to wounding. TSPs are likely to contribute to post-injury tissue repair and remodeling [21]. In this review, we consider structural and functional aspects of the TSP protein family in relation to cardiovascular disease.

## 2. Thrombospondins Structure

**Figure 1:**
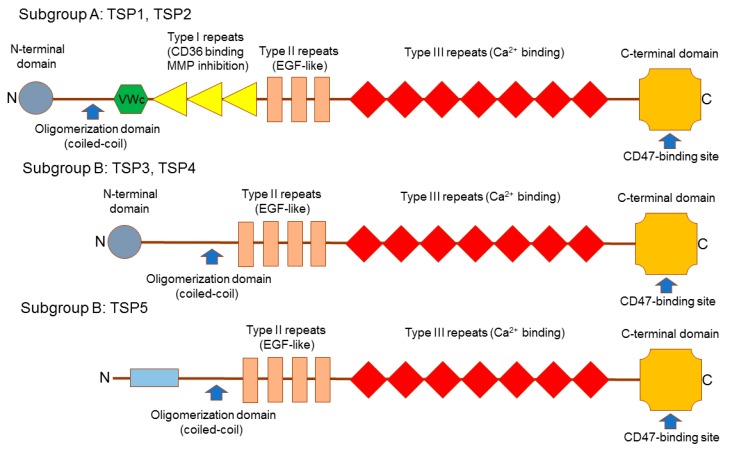
Structure of thrombospondins (TPSs). TSP family includes five members: TSP1–5. Subgroup A comprises TSP1 and TSP2 that form pentamers while subgroup B contains trimeric TSP3–5. TSPs have a complex multidomain architecture that provides an option to bind various ligands. For example, C-terminal domain contains CD47-binding site. Type III repeats are involved in Ca^2+^ binding while Type I repeats are responsible for the interaction with CD36, a receptor for TSP1 and TSP2, and inhibition of matrix proteinases (MMPs). Type II EGF-like domains are involved in the regulation of various signaling pathways such Notch and others. **C**-terminal domain, Type III repeats and Type II epidermal growth factor (EGF)-like repeats share high homology in all TSPs and represent a signature of the TSP family. Von Willebrand factor type C (VWc) domain is cysteine-rich and is implicated in binding members of the transforming growth factor-β (TGF-β) superfamily. The oligomerization (coiled-coil) domain drives formation of TSH homooligomers. The N-terminal domain, which is present in TSP1–4 and absent in TSP5, is less conservative. This domain regulates structure and stability of the coiled-coil region and binds heparin.

The TSP family contains five members (TSP1–5) that represent multimeric glycoproteins, which bind Ca^2+^, interact with other ECM proteins, and contribute to the associations between cells and between cells and ECM. TSMs divided to two subgroups: trimeric subgroup A (TSP1 and TSP2) and pentameric subgroup B (TSP3, TSP4, and TSP5). TSPs have a complex multidomain structure (Figure 1). The C-terminal domain, type III repeats and epidermal growth factor (EGF)-like repeats are present in all TSPs and underline the TSP family. The oligomerization domain can be also found in all family members but it is more variable compared with other shared structures [22]. The subgroup A has three EGF-like repeats and type I repeats (also known as thrombospondin repeats; TSRs), von Willebrand factor type C (vWC) domains, and the N-terminal domain. The subgroup B contains four EGF-like repeats but vWC domains and TSRs are missing. While TSP3 and TSP4 have the N-terminal domain, TSP5 has not [23].

The evolutionary analysis showed that the subgroup B is evolutionary younger than the subgroup A [24]. In fish, a TSP4-like sequence, an ortholog of the tetrapod *TSP5* gene is found [25]. The TSP5-coding sequence evolved more quickly from the TSP4-like sequence as an innovation in the tetrapod lineage. Thus, all *TSP* genes show conservation of synteny between fish and tetrapods. In humans, the *TSP1*, *TSP3*, *TSP4* and *TSP5* genes reside within paralogous regions and are the result of gene duplications [24].

Due to the availability of various structural domains, TSPs can interact with different surface receptors and ECM proteins. In the TSP molecule, EGF-like domains are employed for binding of integrins and Ca^2+^, TSPs are needed to bind transforming growth factor (TGF)-β and CD36, while the N-terminal domain is required for binding heparin and integrins [26]. The C-terminal domain contains a binding site for CD47, an essential TSP receptor [27]. TSP-mediated effects indeed depend on the availability of the binding partner and local microenvironment that explains cell- and tissue-specific actions of TSPs. In addition, TSPs display different cellular distributions, different temporal expression profiles and have distinct functional responsibilities and modes of transcriptional regulation.

TSP1 that was identified first represents the most studied thrombospondin. TSP1 and TSP2 are expressed by several cell types in response to damage or during remodeling [28,29]. TSP1 is known due to the functional significance in the control of angiogenesis and thrombosis and capacity to increase bioavailability TGF-β by liberating this cytokine from its latent form [23]. In the heart, TSP2 contributes to maintaining of cardiac matrix integrity via its actions on matrix metalloproteinases (MMPs) [30].

The highest expression levels of TSP3 and TSP5 were detected in the vascular wall and tendon [31]. However, both these TSPs are the least studied among TSP family members. TSP4 has a role in vascular inflammation [31], regulation of myocyte contractility, angiogenesis, and ECM remodeling [32,33].

In our understanding of TSP functions, the most profound recent progress was made in studying the cardiovascular system. Genetic studies showed association between single nucleotide polymorphisms (SNPs) in *TSP1*, *TSP2*, and *TSP4* genes with cardiovascular pathology [34,35,36,37,38,39]. All TSP disease-associated SNPs are functional. For example, the A387P polymorphism of *TSP4* and N700S polymorphism of *TSP1* alter Ca^2+^-binding sites [40]. Ca^2+^ binding is essential for TSP structure and function. The TSP1 S700 variant had significantly less capacity to bind Ca^2+^ compared to the N700 allele. In fact, the P387 variant of *TSP4* represents a gain-of-function allele because it acquired an additional Ca^2+^-binding site absent in the A387 allele [40]. A recent meta-analysis showed that the N700S polymorphism of *TSP1* is associated with coronary artery disease (CAD) especially in Asian populations (heterozygote model: odds ratio (OR) = 1.57 [95% confidence interval (CI): 1.01–2.44]; dominant model: OR = 1.56 [95% CI: 1.00–2.43]). The *TSP4* A387P polymorphism is associated with increased CAD risk in American population (homozygote model: OR = 1.29 [95% CI: 1.04–1.61]; recessive model: OR = 1.27 [95% CI: 1.02–1.58]). No association was shown for the THS2 3′ (untranslated region) UTR polymorphism and higher CAD risk [41].

## 3. Cardiac Integrity

Expression of all TSP family members was found in the heart. In cardiac remodeling, TSP1, TSP2, and TSP4 are up-regulated [42,43,44,45]. In pressure overload, myocardial expression of TSP3 and TSP5 was shown to be also increased [46].

TSP1, TSP2, and TSP4 are involved in cardiac fibrosis but possess opposite effects. While TSP1 and TSP2 promote fibrosis [47,48], TSP4 inhibits profibrotic mechanisms as was shown in animal models [49,50] and human heart allografts [43,48]. In human allografts, increased levels of TSP1 and TSP2 suggested for induction of both the fibrotic response and allograft rejection [43,48]. In TSP1 or TSP2-deficient murine cardiac remodeling models (i.e., those affected with doxorubicin-induced cardiomyopathy [51], diabetic cardiomyopathy [52,53], dilated cardiomyopathy [54], or MI [44,55,56] the profibrotic role of both TSPs was confirmed. Mechanistically, profibrotic effects of TSP1 and TSP2 in the heart lead to the stimulation of TGF-β, a key inducer of cardiac fibrosis [48,57,58], suppression of MMPs [48,59], and inhibition of angiogenesis [43,47,55]. Profibrotic activity of TSP1 can be also mediated the calreticulin/low density lipoprotein receptor-related protein 1 (LRP1) complex whose stimulation results in the activation of prosurvival protein kinases such as PI3K and migration of fibroblasts [60,61]. The N-terminal domain of TSP1 has the calreticulin-binding site to stimulate association of calreticulin with LRP1 to the signal focal adhesion disassembly and providing signal into the cytoplasm [62].

TSP2 expression was observed only in biopsy specimens from the hypertrophic hearts of rats that rapidly developed heart failure suggesting for a possible value of TSP2 to serve as a marker of early onset of heart failure [59]. TSP2-deficient mice were extremely vulnerable to rapid progression from angiotensin II-induced cardiac hypertrophy to cardiac failure and fatal rupture since 70% of animals died from cardiac rupture whereas the rest of them progressed to heart failure [59]. These data therefore indicate a role of TSP2 as an essential regulator of cardiac integrity.

In the heart, lack of TSP4 leads to advanced fibrosis [49,50] suggesting for the anti-fibrotic role. TSP4 also triggers heart stress adaptation by increasing intracellular Ca^2+^ content in cardiac muscle cells and enhancing contractility [63]. TSP4-induced adaptive response against ER stress also protects cardiomyocytes from pressure overload [1]. In TSP4-deficient mice, tendon collagen fibrils were found to be significantly larger than in wild-type mice suggesting for the negative TSP4-dependent regulation of collagen synthesis in ligaments [64]. However, it is unknown whether this TSP inhibit cardiac collagen production.

The reason of profound differences in the actions of TSP4 and TSP1/TSP2 on myocardial fibrosis may rely on the structural differences between these TSPs. TSP4 lacks domains responsible for the control of MMP function, angiogenesis and TGF-β stimulation. These domains are present in TSP1 and TSP2 [23]. Thus, in order to recognize effects of every TSP in heart remodeling, it is necessary to monitor expression of each TSP in various steps of remodeling and adaptive reaction to heart damage.

Expression of TSP1–4 was found in human aortic valves [65]. In fibrosclerotic and stenotic valves, TSP2 production was increased. TSP2 was up-regulated in myofibroblasts and some endothelial cells (ECs) and was associated with myofibroblast proliferation and neovascularization. TSP2 activation was followed by suppression of Akt and NF-κB [65]. Cardiac-related expression of TSP4 was highest in the valves suggesting for a potential activity in these regions [32,50].

## 4. TSPs in Angiogenesis

Angiogenesis is an essential physiological process involved in the developmental vasculogenesis and tissue repair after injury. In pathology, formation of neovessels occurs in vascular proliferative diseases such as atherosclerosis and in tumors. The anti-angiogenic properties of TSP1 and TSP2 are established and confirmed in different models of angiogenesis [66,67]. The anti-angiogenic activity of TSP1 and TSP2 may be especially attractive in the context of anti-tumor therapy because both TSPs are able to inhibit tumor-associated angiogenesis and suppress tumor growth [68,69]. Studying of TSP1 effects on tumor neovessel formation provided new insights into a specific role and a power of this TSP in preventing angiogenesis. It became obvious that tissue expression of TSP1 is able to define fate of angiogenesis even without influencing by pro-angiogenic signals [70].

A phenomenon of dormant tumors that have a microscopic size and do not expand is associated with high expression and inhibitory effects of TSP1 and tissue inhibitor of matrix proteinases TIMP-1 [71]. Down-regulation of TSP1 and reduced tumor sensitivity to angiostatin leads to proangiogenic switch and induction of rapid growth and tumor expansion [72]. MicroRNA (miR)-467 was reported to function as a negative regulator of TSP1 expression [73]. This miRNA is induced by high glucose and leads to the sequestration of TSP1 mRNA in the non-polysomal fraction of tumor cells and induction of angiogenesis. Inhibition of miR-467 suppresses tumor growth and angiogenesis [74].

The anti-angiogenic activity of TSP1 is attributed to a structural domain known as the TSP type I repeat [75]. This domain serves as a single Ca^2+^-binding site for endothelial receptor CD36 that is essential for mediating anti-angiogenic effects of TSP1 and TSP2 [76,77]. Histidine-rich glycoprotein (HRGP), a circulating protein, contains a CD36 homology domain and blocks TSP-dependent anti-angiogenic effects by binding to either TSP1 or TSP2 [75,78]. Another TSP-1-dependent anti-angiogenic mechanism is consisted of the engagement of CD47 that disrupts CD47 interaction with vascular endothelial growth factor (VEGF) receptor 2 (VEGFR2) and blunts VEGFR2-mediated proangiogenic signaling associated with activation of endothelial NO synthase (eNOS) and soluble guanylate cyclase (sGC) [79]. In silico analysis showed that TSP1 binding to CD47 can also enhance VEGFR2 degradation [80]. By contrast, in TSP1-deficient mice, phosphorylation of endothelial VEGFR2 is up-regulated thereby providing a stimulatory signal to Akt or Src that in turn activate eNOS through phosphorylation [79]. Another suppressive mechanism, by which TSP1 can inhibit myristic acid-stimulated eNOS-dependent signaling that leads to the induction of increased adhesion properties of ECs and vascular smooth muscle cells (VSMCs), is dampening of the CD36-mediated uptake of free fatty acids or engagement of CD47 [81,82].

TSP1 can also diminish the sGC/3′, 5′-cyclic GMP (cGMP) signaling by limiting cGMP-dependent activation of the downstream cGMP-activated kinase (PKG) [83]. The inhibitory effect of TSP1 on eNOS/cGMP signaling in ECs are more potent than that of TSP2 suggesting for a role of TSP1 as a dominant regulator of NO/cGMP signaling pathway through CD47 [84].

NO is essential for activation sGC that contains a heme responsible for NO binding [85]. NO-dependent stimulation of sGC leads to intensive production of cGMP, a signaling messenger that is involved in the vascular tone regulation by relaxation of VSMC contractility [86], inhibiting platelet aggregation [87] and blood cell adhesion to the endothelium [88]. Except for limiting NO bioavailability, inhibitory effects of TSP1 on sGC activity and cGMP production can also involve suppression of the hydrogen sulfide (H_2_S)-mediated signaling through blocking activity of H_2_S-biosynthesiting enzymes, cystathionine β-synthase (CBS) and cystathionine γ-lyase (CSE) [89]. Another inhibitory mechanism may involve TSP1-dependent stimulation of reactive oxygen (ROS) and nitrogen species production [90], which in turn inactivate sGC via covalent enzyme modification [91] or heme nitrosylation [92].

Compared to TSP1 and TSP2, TSP4 exerts proangiogenic properties [93]. TSP4 was detected in the lumen of neovessels. TSP4-deficient mice have diminished angiogenesis in comparison with wild-type mice. Mice transgenic for the CAD-associated human *TSP4* P387 variant displayed more intensive angiogenesis compared with mice bearing the A387 allele of *TSP4*. Pulmonary ECs derived from TSP-lacking mice exhibited reduced adhesion and migratory properties in contrast to wild-type ECs. In addition, recombinant TSP4 was shown to stimulate vessel development and EC motility/proliferation through binding to integrin-α2 and gabapentin receptor α2δ-1 [93].

## 5. TSPs in Atherosclerotic Blood Vessels

Vascular expression was shown for all TSPs [31]. In blood vessels, TSP1, TSP2, and TSP4 are involved in the functional and structural regulation of the vascular wall and interactions with blood-borne cells. In diabetic rats, TSP1 levels were increased after vascular wounding [94]. In rats with balloon-induced injury, blockade of TSP1 in carotid artery with antibody resulted in enhanced reendothelization and decreased neointima formation [95]. In apolipoprotein E (ApoE)-deficient mice, an atherosclerotic animal model, inhibition of TSP1 and TSP4 caused delayed atherosclerosis progression and less inflammatory conditions. However, at late atherogenic stages, the plaques developed proinflammatory content and had the same size as lesions in control mice [31,96]. TSP-1 or TSP4 did not alter intraplaque lipid content but induced dramatic changes in macrophages counts within the plaque by influencing either function (TSP1) or infiltration (TSP4) of macrophages to the lesion. In advanced lesions, TSP2 plays an anti-atherogenic role by stimulating phagocytic function of macrophages needed to perform clearance of apoptotic and necrotic cells and cell debris [96].

In ApoE-deficient mice, TSP4 depletion does not affect lesional matrix deposition [31] while TSP1 deficiency leads to the formation of more fibrotic lesions of less size but also increases inflammation associated with enhanced activity of macrophages [96]. These macrophages are involved in intensive degradation of the fibrous cap associated with increased accumulation of MMP-9 in the cap but display altered phagocytosis that finally results in increase of the necrotic core and plaque destabilization. It appears that TSP1 promotes atherogenesis in early stages through induction of endothelial dysfunction, stimulation of VSMC proliferation and inhibiting collagen deposition. However, in late stages, TSP1 switches this role to the anti-atherogenic function by repressing lesional maturation via stimulation of the phagocytic activity of macrophages and reducing necrosis [97].

In ApoE-deficient mice fed on high-fat diet, strong plaque TSP1 expression was detected in fibrous cap-associated VSMCs and inflammatory cells in the shoulder of the plaque and foam cells. Weak TSP1 expression was found in the adventitia and media of the atherosclerotic wall [96]. Expression of TSP2 was found in human arterial VSMCs [98] but was not detectable in the endothelial plaque lining and intraplaque neovessels [99]. TSP3, TSP4, and TSP5 are all also linked to atherosclerotic plaques. Expression of TSP3 was detected in the tunica media and tunica adventitia, on the luminal endothelial surface, and in the plaques in ApoE-deficient mice [31]. TSP4 exerts proatherosclerotic and proinflammatory effects in vessels since TSP4-knockout mice developed less inflammatory lesions with lowered macrophage content, diminished activation of ECs, and reduced production of proinflammatory cytokines [31]. TSP4 stimulates adhesion and movement of macrophages and neutrophils in an integrin α_v_β_3_-dependent manner [13,40]. The CAD risk-associated *TSP4* P387 variant was shown to enhance leukocyte attachment to ECs and motility and promote proinflammatory signaling in vascular and blood-borne cells likely due the ability to bind more Ca^2+^. This ability leads to conformational changes in the mutant TSP4 molecule and provides better interaction with cell surface receptors [13,40].

TSP5 protein was histochemically detected in normal and affected (i.e., atherosclerotic and stenotic) human arteries where it is produced by VSMCs [100]. In ApoE-deficient mice, TSP5 expression is associated with tunica media and a few plaque cells [31]. TSP5 is involved in maintaining VSMC quiescence and contractile phenotype via interaction with integrin α_7_β_1_ [101]. A disintegrin and metalloproteinase with thrombospondin motifs 7 (ADAMTS7) that is also expressed in VSMCs can degrade TSP5 and promote VSMC migration and recruitment for neointima formation [102].

CD47 (also known as the integrin-associated receptor; IAP) is the receptor for TSP1 [103]. After stimulation with TSP1, CD47-mediated pathway is involved in the control of leukocyte functions, vascular resistance, and intracellular signaling in ECs and VSMCs [104]. TSP1 binding to CD47 has global functional consequences by inhibiting endothelial nitric oxide (NO) production, controlling vascular tone, and maintaining systemic hemodynamics and cardiac dynamics in stressful conditions [105,106,107,108]. The interaction between TSP1 and CD47 also regulates thrombosis/hemostasis, immune responses, and mitochondrial function [109]. CD47-binding capacity can be shared between all TSP family members since CD47-binding site is located in the C-terminal region, which is homologous in all TSHs.

TSP1-mediated overactivation of NADPH oxidase may exert important pathogenic effects in cardiovascular pathology. TSP1 is able to stimulate Nox1 and Nox4 through CD47-dependent signaling. In VSMCs, TSP1 binding to CD47 leads to phospholipase C (PLC)-catalyzed biosynthesis of diacyl glycerol, a stimulator of protein kinase C (PKC) that in turn phosphorylates the NADPH oxidase core subunit p47^phox^ followed by activation of Nox1. Nox1 overactivity enhances ROS formation that further inhibits VSMC-dependent vasorelaxation and induces vascular dysfunction by promoting oxidative stress [90]. In ischemic VSMCs, TSP1 can also increase ROS generation via stimulation of the cell surface receptor signal-regulatory protein-α (SIRP-α) and subsequent recruitment of the p47^phox^ subunit [110].

TSP1-induced Nox4 up-regulation stimulates ROS-dependent proliferation of VSMCs and neointimal formation, a hallmark of the proatherogenic arterial remodeling [111,112]. In addition, TSP1/CD47-dependent stimulation of Nox1 enhances micropinocytosis of non-modified low density lipoprotein (LDL) by macrophages and promotes their transformation to foam cells, another key characteristics of atherogenesis. In macrophages, Nox1 overactivity induces dephosphorylation of actin-binding protein cofilin, PI3K-dependent activation of myotubularin-related protein 6 (MTMR6) followed by cytoskeletal rearrangements and increased LDL uptake [113]. Thus, TSP1-mediated stimulation of ROS-dependent signaling and oxidative stress have numerous pathogenic consequences, which promote atherogenesis.

## 6. TSPs in Myocardial Infarction

Myocardial injury initiates the post-MI tissue repair response aimed to restore cardiac conduction and contractility, blood supply, and replace necrotic cardiomyocytes in the infarct with a scar [114]. After MI, cardiomyocyte necrosis occurs early in post-MI remodeling of the infarct area while apoptosis occurs in the infarct and distant cardiac regions both in the early and late stages of remodeling [115]. In the early stage of cardiac repair, infiltrated inflammatory cells release MMPs, particularly MMP-2/9, to degrade ECM in the injured myocardial area and adjacent regions [116]. In parallel with ECM destruction, transformation of cardiac fibroblasts to myofibroblasts associated with their proliferation and migration to the site of injury begins. In response to profibrotic signals, myofibroblasts produce collagen and other ECM components, which are deposited in the cell-free infarcted zone that was cleared by macrophages from necrotic cells [117]. In the proliferative stage of myocardial repair, the collagen amount quickly rises in the injured region while collagen fibers undergo cross-linking in the maturation stage [118]. Collagen can be also accumulated in the non-infarcted area that can initiate reactive myocardial hypertrophy [116].

Compared to pressure overload, post-MI reperfusion in TSP1-deficient mice leads to more intensive and long-term heart inflammation in the infarct border zone and excessive remodeling [55]. These observations allow to suggest that TSP1 exerts a barrier function in the infarct border zone to limit propagation of inflammation and fibrosis into the non-injured cardiac regions. The mechanisms of this effect are not well studied. TGF-β-dependent up-regulation of TSP1 production may be involved in this process [55,56]. TSP1 also enhances apoptosis of activated T cells through CD47-dependent activation of proapoptotic Bcl-2 family member BNIP3 that primarily links it to inflammation [119,120].

TSP1 inhibits both eNOS-dependent NO production and NO-mediated signaling that stimulates vascular relaxation and angiogenesis [107]. These data may suggest for a putative contribution of TSP1 to heart ischemia and/or MI [121]. However, TSP1 also exhibit cardioprotective effects by activating TGF-β, an anti-inflammatory cytokine [56]. Indeed, inhibition of CD47 represents a more attractive therapeutic target than inhibition of TSP1. In ischemic mouse models, exposure to CD47-blocking agents results in significant improvement of tissue survival and decreased vasculopathy, an evidence of the therapeutic value of CD47 suppression to treat cardiovascular disease [122]. In MI or ischemia, down-regulation of CD47 may have unpleasant sequela such as diminished apoptosis of inflammatory macrophages, which can result in elevated levels of proinflammatory cytokines [123]. Enhanced TSP1 production may be involved in NO resistance observed in aging and ischemic heart disease [122]. Indeed, therapeutic targeting of vessel NO signaling through TSP1/CD47 can be valuable [121].

Compared with TSP1, our knowledge of a role of TSP2 in MI is constrained. TSP2 and TSP4 do not involved to the control of NO signaling [106]. However, TSP2 shares with TSP1 many anti-angiogenic properties, promotes CD36-mediated apoptosis of ECs, and initiates cell cycle arrest [68]. TSP2-deficient mice exhibited increased angiogenesis that was associated with MMP-9 up-regulation [123]. Similarly, TSP2 deficiency was shown to induce enhanced angiogenesis and delayed skin wound contraction accompanied with increased MMP-2/9 and soluble VEGF production [124]. These findings indicate that anti-angiogenic effects TSP2 can be released through multiple mechanisms. TSP2 deletion in mouse was shown to induce increased vascularity and defects in connective tissue formation due to impaired collagen fibrillogenesis [125,126] indicating that cardiac TSP2 may be involved in post-injury heart remodeling and repair through the control of fibrillogenesis [127]. In favor of the involvement of TSP2 into cardiac repair, the ability of TSP2 to positively modulate function of human cardiomyocyte progenitor cells (hCMPCs) in hypoxic conditions was demonstrated [128]. In mice, short-term exposure to hypoxia stimulates migratory and invasive properties of hCMPCs while prolonged exposure activates proliferation, angiogenesis, and blocks migration likely due to TSP2 actions [129]. Limitation of migratory activity of hCMPCs is necessary to induce proliferation and differentiation of progenitor cells into cardiomyocytes in the infarcted region.

Post-MI and in cardiac hypertrophy, TSP4 expression was shown to be chronically up-regulated in the heart, especially in the left ventricle [45]. TSP4 mRNA levels directly correlated with the rate of left ventricular remodeling indicating the role of TSP4 in post-MI cardiac remodeling [130]. TSP4 plays a cardioprotective role by diminishing cardiac ER stress. Furthermore, cardiac TSP4 overproduction is protective against MI [63]. Further studies showed that the type III repeat domain and the C-terminal domain of TSP2 are involved in Atf6α binding and regulation of ER stress response [131]. ATF6α is an ER stress-regulated transcription factor that drives expression of ER chaperons needed to initiate the unfolded protein response and prevent ER stress [132]. TSP4 cardiac-specific transgenic mice was resistant to myocardial infarction (MI) while TSP4-deficient mice exert cardiac maladaptation.

In the pilot genetic study, an association of the *TMP1* N700S and *TMP4* A387P variants with higher risk familial premature MI was demonstrated. The *TMP2 T/G* 3′UTR polymorphism was associated with lower risk of familial MI [34]. A global meta-analysis confirmed association with CAD only for the *TMP1* N700S and *TMP4* A387P polymorphisms but not for the *TMP2 T/G* 3′UTR [41]. The *TSP4* A387P polymorphism was associated with increased coronary risk in post-MI subjects who had elevated levels of high density lipoprotein (HDL) cholesterol and C-reactive protein (CRP), an inflammatory marker [39]. Accordingly, the TT genotype of the *TMP2 T/G* 3′UTR variant showed association with plaque erosion independently of age, gender, and cigarette smoking in cases of sudden death [133]. Lesional erosion that is induced by intimal injury and does not lead to plaque rupture is a frequent cause of sudden death [134]. However, the results of case-control studies were contradictory since no significant association of the *TMP1* N700S, *TMP2 T/G* 3’UTR, and *TMP4* A387P genetic variants, and with both CAD and MI were found in other studies [135,136,137,138]. Possible reasons of such an inconsistency may be referred to the different patients’ selection criteria, insufficient size of the population samples tested, racial differences, etc. For example, the frequency of the *TSP1* N700S variant was reported to be extremely low in the Chinese Han population [138]. This therefore underlines the need to recruit case-control cohorts of a larger size to provide a sufficient statistical power that is critical to the success of genetic association studies to detect causal genes of human complex diseases such as CAD.

## 7. TMPs in Cardiac Hypertrophy

Cardiac hypertrophy can be induced by chronic pressure overload (for instance, by essential hypertension) and is characterized by extensive growth of cardiac muscle cells, proliferation of cardiac fibroblasts, increased ECM deposition (i.e., fibrosis), and intensive cell death. Heart fibrosis occurs due to the massive production and deposition of collagens type I and type III, which exceeds their degradation. Fibrosis is resulted from the up-regulation of collagen synthesis, down-regulation of collagen destruction, or both [139]. In heart hypertrophy, cardiac matrix composition is altered due to collagen redistribution and increased cross-linking that can lead to changes in ECM functional properties [140]. MMPs is the most frequent type of enzymes involved in matrix remodeling. Except for matrix degradation, MMPs also promote ECM synthesis by liberating growth factors and other profibrotic messengers from the matrix [141]. In the heart, chronic hypertension stimulates apoptosis of cardiomyocytes [142] and inflammation [143].

In hypertensive cardiac disease, levels of TSP1, TSP2, and TSP4 are elevated [45,59,144]. Pressure overload stimulates heart expression of TSP1 and TSP4 [45,144]. TSP2 up-regulation was observed in hypertrophic hearts of rats that overexpressed renin and further progressed to heart failure [59].

In mice, TSP1 deficiency led to early onset of heart hypertrophy and enhanced late dilatation in response to pressure overload. Degenerative morphological changes in cardiomyocytes were observed due to the sarcomeric loss and rupture of sarcolemma. Cardiac remodeling was abnormal and accompanied with abundant infiltration of defective fibroblasts. The fibroblast-to-myofibroblast transdifferentiation was impaired. Collagen synthesis was reduced due to the perturbed TGF-β signaling. Furthermore, myocardial production of MMP-3 and MMP-9 was up-regulated [144]. Indeed, TSP1 loss in the heart leads to adverse consequences by impairing response to pressure overload and inducing aberrant tissue remodeling. TSP1 activation in the pressure-overloaded myocardium is critical in the control of the fibroblast phenotype and heart remodeling through up-regulation of TGF-β-dependent pathway and cardiac matrix preservation through inhibition of MMPs. However, no significant changes in inflammatory responses was detected [144] that is rather paradoxical since TGF-β exerts anti-inflammatory properties. In an ischemia-reperfusion model, TSP1 deletion was associated with prolonged post-MI inflammatory response and increased release of proinflammatory factors such as chemokine (C-C motif) ligand 2 (CCL2), chemokine (C-X-C motif) ligand 10 (CXCL10), interleukin (IL)-1β, IL-6, macrophage inflammatory protein-1α (MIP-1α) [55]. In experimental diabetic cardiomyopathy complicated with abdominal aortic coartaction, implementation of LKSL, a peptide that antagonizes TSP1-dependent TGF-β activation, had beneficial actions on the myocardium by inhibiting TGF-β-driven fibrosis [52]. Hence, from the pharmacological point of view, antagonizing TSP1-dependent activation of TGF-β looks more attractive and efficient than blockade of TSP1 signaling in heart hypertrophy.

Reduced vascularity because of decreased angiogenesis is supposed to promote progression of heart hypertrophy to heart failure [145]. The inhibitory actions of TSP1 on cancer angiogenesis were broadly investigated. However, there are contradictory results of studies that examine microvascular effects of TSP1 in the heart. Global deletion of TSP1 was reported to induce the development of dense cardiac capillary network and higher cardiac mass [146]. These findings were not confirmed [144]. Up-regulation of MMPs in TSP1-deficient mice may substantially contribute to enhanced angiogenesis [144].

In response to vasoactive stress, TSP1-deficient mice responded by increased heart rate and changes in blood pressure by elevation of central diastolic and mean arterial blood pressure and reduction of peripheral blood pressure and pulse pressure. In response to epinephrine, the hypertensive response was diminished in both TSP1-deficient or CD47-deficient mice [105]. These data indicate an important role of TSP1 and its receptor CD47 in the acute regulation of blood pressure, which possess vasoconstrictor effects to hold global hemodynamics under vasoactive stress. By inhibiting NO-dependent vasorelaxation, TSP1 maintains blood pressure under stressful conditions [107]. Since CD47 is crucially involved in TSP1-mediated regulation of blood pressure, pharmaceutical targeting of the TSP1/CD47 mechanism may be useful for treatment of hypertension.

In cardiac hypertrophy, TSP2 and TSP4 activities are rather oriented to the control of matrix composition. In pressure overload, TSP2-lacking mice developed cardiac rupture or heart failure accompanied with higher activities of MMP-2/9 indicating that TSP2 contributes to the control of cardiac integrity in hypertrophic hearts [59]. The up-regulation of TSP2 was recognized as a useless effort to rescue the integrity in pressure overload. In response to experimentally induced transverse aortic constriction (TAC), TSP4-deficient mice exhibited advanced heart hypertrophy and fibrosis along with left ventricular dilation, decreased systolic and impaired diastolic function. These cardiac changes resembled age-dependent hypertrophy and fibrosis [50]. TAC-induced interstitial fibrosis was accompanied with up-regulated collagen production, increased MMP expression, lowered microvessel density and was not associated with apoptosis of cardiac muscle cells and inflammation [147]. However, it is unclear whether decrease in myocardial capillary density is the effect of fibrosis or angiogenesis.

TSP4-lacking hearts failed the ability to respond properly to acute pressure overload by enhanced heart contractility or by activation of stretch-response pathways (Akt- and ERK1/2-dependent). In addition to missing capacity to reply normally to acute pressure overload, TSP4-deficient mice also failed the ability to perform an appropriate adaptive response to chronic press overload that induces cardiac dilation, greater myocardial mass, and decline in heart function. However, no changes in interstitial fibrosis was detected. Pressure overload affected cardiac contractility of a whole cardiac muscle, not in separate cardiomyocytes [49]. Therefore, TSP4 can serve as a cardiomyocyte-interstitial mechano-sensing molecule, which regulates adaptive myocardial contractile reactions in response to acute stress. Stable and stressed ECM is likely to negatively regulate function of cardiac muscle cells, which is confronted by TSP4 in normal conditions [49].

## 8. TSPs in Heart Failure

The aberrant remodeling leads to myocardial overwork that if untreated can progress to heart failure. Structural changes associated with right or left heart failure are differentiated [148]. Right failure is characterized by intensive collagen degradation and cross-linking disruption and off-center hypertrophy. In left failure, cardiomyocyte hypertrophy is concentric while interstitial collagen content and cross-linking is increased [149]. Heart remodeling is accompanied by substantial cardiomyocyte loss, which is supposed to represent one of the major mechanisms of heart failure progression. Apoptosis, necrosis, and autophagy mediate the cardiomyocyte death [150,151]. Cardiac inflammation also contributes to heart failure through enhanced production of TNF-α and other inflammatory cytokines that exhibit adverse effects on the myocardium [152].

TSP1 levels were decreased in human failing hearts and positively correlated with TGF-β levels indicating that cardiac fibrosis is an attribute of early stages of heart failure [153]. In rats with artificially induced heart failure, TSP1 production was up-regulated [154,155]. The TSP1/CD47 signaling seems to play a central role in promoting left ventricular hypertrophy and heart failure [155]. Up-regulation of histone deacetylase 3 (HDAC3) and Ca^2+^/calmodulin protein kinase II (CaMKII) stimulates hypertrophy of the left ventricle. Accordingly, blockade of either CD47, HDAC3 or CaMKII has beneficial cardiac effects by reducing hypertrophy and softening heart failure [155] thereby underlining a value of the TSP1/CD47 pathway in the pathogenesis of heart failure.

In a murine model of age-related heart failure, a modulatory role was shown for miR-18/19, both are members of the aging-associated miRNA cluster 17–92 that targets TSP1 and connective tissue growth factor (CTGF) [156]. In age-related heart failure, expression of cluster 17–92 miRNA members was down-regulated. Accordingly, TSP1 and CTGF were increased. Importantly, these expression changes occurred only in cardiomyocytes, not in fibroblasts. Indeed, miR-18/19 protect heart against age-related heart failure. With aging, expression of the cluster 17–92 declines while expression of TSP1 and GTCF grows, a phenomenon that predisposes to heart failure [156].

In aged mice, TSP2 production is also increased and is attributed to the ECM surrounding cardiac muscle cells [54]. In older animals, TSP2 deficiency was associated with severe dilated cardiomyopathy, cardiac fibrosis, altered systolic function, and inflammation. Cardiomyocytes were subjected to increased cellular stress and death. Cardiac capillary density was not affected. MMP-2 expression was up-regulated and tissue transglutaminase-2 was down-regulated that led to aberrant cross-linking. Cardiac expression of the TSP2 transgene prevented dilated cardiomyopathy in aged rats [54].

The cardioprotective action of TSP2 was confirmed in a model of doxorubicin-induced cardiomyopathy. Deletion of TSP2 in mice caused higher death rate in response to doxorubicin while survived animals has diminished heart function associated with intensive apoptosis of cardiac muscle cells and ECM destruction because of MMP-1/9 activation [51]. As shown in a model of viral myocarditis-induced heart failure, TSP2 deficiency also stimulates heart inflammation suggesting for the involvement of TSP2 in the regulation of inflammatory response [157]. Lack of TSP2 is related to reduced activation of regulatory T cells, advanced necrosis and fibrosis, heart dilation, and diminished systolic function. TSP2 overexpression prevents heart failure and reduces mortality through attenuating heart inflammation, inflammation, and virus-induced cardiac death. TSP2 was also up-regulated in the myocardial biopsy samples from subjects affected with viral myocarditis [157]. Overall, these data demonstrate protective effects of TSP2 against heart failure.

The up-regulation of cardiac TSP4 expression was observed in the heart of rats with pressure overload-induced heart failure [45,158] and in rats with heart failure induced by the volume overload after aortocaval fistula [159]. Interestingly, there were no significant changes in systolic function and expression of genes responsible for Ca^2+^ homeostasis, neurohumoral regulation, contractility, and cytoskeleton organization during transition from left ventricular hypertrophy to heart failure [158]. Only, expression of ECM proteins such as TSP4 and matrix Gla protein was elevated indicating that progression from hypertrophy to heart failure is regulated by ECM remodeling. In the heart of TSP4-deficient mice subjected to TAC to increase left ventricle load, massive ECM depositions, higher cardiac mass, decreased microvessel density, abnormal heart function, and inflammation, but no signs of apoptosis were observed [50]. These observations show that increase of cardiac TSP4 expression is an adaptive response to pressure overload. TSP4 display cardioprotective effects by regulating myocardial remodeling in pressure overload to prevent progression to heart failure.

## 9. TSPs in Calcific Aortic Valve Disease

The ECM and factors that control ECM composition and remodeling are implicated in the pathogenesis of calcific aortic valve disease (CAVD). Inflammation and angiogenesis, both are regulated by TSPs, also closely linked to the pathogenesis of aortic stenosis [160]. CAVD is characterized by increased proliferation of myofibroblasts, neovasculogenesis, and valvular calcification. Expression of TSPs 1–4 was detected in stenotic valves, with up-regulation of TSP2 [65]. TSP2 expression was permanently increased in neovessels during progression from early valve remodeling to adverse stenosis indicating a role of TSP2 in the control of CAVD-associated neovascularization [65]. Further studies are required to discover a precise mechanism underlining a role of TSP2 in calcified aortic valves.

## 10. TSPs in other Pathologies

The involvement of TSP1 to hypoxia-induced pulmonary hypertension was shown. This disorder is characterized by increased pressure in the pulmonary artery, pulmonary vein, and lung vasculature. Pulmonary hypertension is accompanied by narrowing of lung-associated vessels due to thickening of the tunica intima and tunica media as a result of pathogenic vascular remodeling. Increased workload of the heart causes hypertrophy of the right ventricle that can finally progress to the right heart failure [161].

Chronic lung ischemia induces overexpression of TSP1 in the pulmonary artery as was shown in a pig model of experimental pulmonary hypertension. TSP1 overactivity correlated with increased death of ECs and endothelial dysfunction likely due to the proapoptotic effects of TSP1 [162]. Similarly, up-regulated levels of various matricellular proteins including TSP1, TSP2, and TSP4 were detected in the right ventricle of monocrotaline-induced pulmonary hypertensive rats [163]. In humans affected with pulmonary hypertension, elevated levels of circulating TSH1 were also reported [164]. Deletion of TSP1 in mice was related to increased arterial VSMC hyperplasia, proliferation, and growth, less advanced vascular remodeling, lowered right ventricular hypertrophy and right ventricle systolic pressure compared with wild-type counterparts exposed to chronic hypoxia. In fact, TSP1-deficient animals showed increased resistance to hypoxia-induced pulmonary hypertension [165].

Mechanistically, TSP1-induced activation of TGF-β promotes VSMC hyperplasia and proliferation in the pulmonary artery and lung arteries of less caliber [166]. In hypoxia-induced human pulmonary artery VSMCs, TSP1 activation also up-regulates production of the NADPH oxidase subunit, Nox4, that can be inhibited by the peroxisome proliferator-activated receptor γ (PPARγ) or its agonist, rosiglitazone [167]. In hypoxic VSMCs, TGF-β acts in an autocrine manner stimulating insulin-like growth factor binding protein-3 (IGFBP-3) activation via the Akt/PI3K mechanism. IGFBP-3 then increases Nox4 expression, which induces VSMC proliferation and transformation of cardiac fibroblasts to myofibroblasts through ROS-dependent signaling and therefore aggravates lung arterial thickening and right ventricular hypertrophy [168,169,170]. Cardiovascular protective effects of PPARγ are mediated through inhibition of hypoxia-induced binding of the transcription factor NF-κB to the *Nox4* promoter that prevents transcription [171]. On the other hand, hypoxia was shown to down-regulate PPARγ in pulmonary arterial VSMCs via the ERK1/2- NF-κB-Nox4 mechanism [172]. In summary, these observations suggest for a pathogenic role of both TSP1 and TGF-β in pulmonary hypertension that cooperate in induction of abnormal tissue remodeling associated with increased arterial VSMC hyperplasia/proliferation and hypertrophy of the cardiac right ventricle.

In addition to the involvement of coronary atherosclerosis and post-MI cardiac remodeling, a role of TSPs in cerebrovascular ischemic disease and ischemic stroke was reported. After stroke, increased production of TSP1 and TSP2 was observed in experimental models of ischemic stroke [173,174]. The post-stroke activation of TSPs is necessary to promote an adaptive response to brain injury in order to the recover synaptic plasticity and motor function [175] and regulate angiogenic and platelet-mediated prothrombotic mechanisms [176]. In the resolution phase of the repair of brain infarct, TSP-1/CD36 interaction is important to activate clearance of dead and apoptotic cells by macrophages in response to stimulation by IL-4 or monocyte colony-stimulating factor (M-CSF) [174,177]. Thus, these data indicate protective effects of TSP1 and TSP2 on brain function during healing of ischemic cerebral injury.

TSP1 plays a protective role in non-ischemic neurological pathology such as Alzheimer’s disease (AD), fragile X syndrome, and Down syndrome, both are associated with serious mental impairments and reduced synaptic plasticity. Decreased expression of TSP1 was shown in a subset of cortical pyramidal neurons and astrocytes that are prone to AD [178,179]. TSP1 was shown to protect neurons against β-amyloid-induced synaptic degeneration [179]. On the other hand, β-amyloid inhibits release of TSP1 by astrocytes that in turn attenuates expression of synaptic proteins such as synaptophysin and PSD95 followed by aberrations in the morphology of dendritic spines and reduction of synaptic plasticity [180]. Similar abnormalities such as spine malformations and reduced synaptic density were observed in Down syndrome astrocytes, astrocytes from animal models of fragile X syndrome, and astrocytes from TSP-deficient mice indicating a pathological role of TSP1 deficits [181,182]. In the AD brain, prostaglandin E2, an inflammatory messenger, reduces astrocytic expression of TSP1 by induction of miR-135 that targets the TSP1 mRNA. Binding of prostaglandin E2 to its receptor EP4 leads to protein kinase A (PKA)-dependent stimulation of the CCAAT/enhancer-binding protein δ (CEBPD), a transcriptional coactivator that up-regulates expression of miR-135 in astrocytes [183]. In summary, these findings suggest for a key role of TSP1 in astrogenesis and maturation, spine development, and synaptogenesis. Thus, decreased TSP1 activity is linked to neurodegenerative, neurodevelopmental, and mental pathology associated with dysfunction of dendritic spines and aberrations in synaptic plasticity.

Thrombospondins may contribute to the pathogenesis of congenital heart defects (i.e., congenital heart disease) associated with structural heart anomalies presented at birth. In children with congenital ventricular septal defect characterized by the perforation of the ventricular septum, serum levels of TSP1 were dramatically increased and showed positive correlation with the risk of ventricular septal defect [184] thereby suggesting for a potential value for early diagnosis of this cardiac defect. A pathologic role of TSP1 up-regulation in the ventricular septal defect is unclear but may be related to the constitutive impairment of the TGF-β signaling associated with alterations of migration of neural crest cells [185] that contribute to the formation of the septum part, which separates the pulmonary circulation from the aorta [186].

Depletion of the Hect domain E3 ubiquitin ligase Nedd4 in mice resulted in detrimental abnormalities in heart development associated with the formation of double-outlet right ventricle and atrioventricular cushion defects that was fatal for developing embryos [187]. TSP1 expression was markedly up-regulated in Nedd4-deficient mice. Nedd4 is involved in the ubiquitination of a variety of ion channels, membrane transporters, growth factors and their receptors [188] followed by proteosomal degradation. Interestingly, VEGFR2 is a Nedd4 substrate [189] whose degradation is promoted by TSP1. Indeed, Nedd4-deficient mice experience adverse problems in cardiogenesis and vasculogenesis associated with letal developmental deviations likely due to the defects in protein trafficking machinery, ER stress, and heart malformations due to enhanced and deregulated growth factor signaling. In addition, Nedd4 deficiency may induce the premature control loss of the activity of sodium channels such as cardiac voltage-gated channel Na_v_1.5 [190] and peripheral neuronal channels Na_v_1.2 and Na_v_1.7 [191], which is rather lethal because of the inability to support heart conduction/contractility and cardiac/neuronal connectivity in a proper manner.

Some cardiac congenital aberrations such as for example the Holt-Oram syndrome are accompanied by alterations in electrical conduction [192]. The role of TSPs in the regulation of myocardial electrophysiology and contraction is widely unclear. Direct evidence for the involvement to the cardiac muscle contractility was obtained only for TSP4 that modulates heart contraction in response to stress induced by enhanced blood flow [49]. In TSP1-deficient mice, no difference in heart contractility was observed compared to the wild-type counterparts [193]. Although TSPs are able to bind many Ca^2+^ cations, this property may be primarily essential for Ca^2+^-dependent signaling. However, there are some evidence in favor of a potential involvement of TPSs in the control of muscle contraction. As mentioned above, the TSP1/CD47 mechanism is implicated in CaKMII-mediated cardiac hypertrophy [155]. In differentiated SMCs, CaKMII controls cell contractility [194]. In peripheral sensory nerves, painful nerve injury interrupts Ca^2+^ signaling by stimulating plasma membrane Ca^2+^-ATPase (PMCA) activity and inhibiting sarco-endoplasmic reticulum Ca^2+^-ATPase (SERCA) activity that results in depletion of ER-associated Ca^2+^ depots and increase of cytoplasmic Ca^2+^ levels. Injury-induced TSP4 up-regulation resembles effects of painful injury on Ca^2+^ homeostasis by reducing Ca^2+^ current (I_Ca_) via high-voltage-activated Ca^2+^ channels and stimulating I_Ca_ through low-voltage-activated Ca^2+^ channels in dorsal root ganglion neurons. This leads to the PMCA activation, SERCA depression, increase of store-operated Ca^2+^ influx, and Ca^2+^ signaling disruption through TSP4-dependent stimulation of the voltage-gated Ca^2+^ channel α_2_δ_1_ subunit (Ca_v_α_2_δ_1_) and PKC-mediated signaling [195]. It would be interesting to examine whether TPSs contribute to the regulation of cardiomyocyte-specific Ca^2+^ handling and activity of SERCA and CaKMII that are primarily involved in the myocardial contractility and electric conductivity.

## 11. Therapeutic Potential of Thrombospondins

The multidomain structural architecture of TSP molecules defines their diverse functions and pleiotropic actions. TSPs have a capability to bind a variety of proteins such as cytokines, growth factors, receptors, and proteases that emphasizes their function in a tissue- and cell type-specific manner [23]. So far, the antiangiogenic activity of TSP1 and TSP2 in cancers inflamed an interest to use these molecules for anti-cancer therapy [196]. Peptides mimicking the anti-angiogenic domains of TSPs and recombinant proteins were developed [197,198].

As known, in TSP1 and TSP2, the anti-angiogenic function is related to the properdin (type-1) repeats located at the N-terminal stalk region [22]. Small peptides derived from this region exhibited only weak inhibitory effects on angiogenesis. However, a single D-amino acid substitution (D-isoleucine) of a particular properdin-region heptapeptide was found to strengthen the anti-angiogenic activity by 1000-fold [199]. Finally, a potent anti-angiogenic TSP1 mimetic nonapeptide analog of this substituted heptapeptide named ABT-510 was constructed [200]. In preclinical studies, ABT-510 showed an ability to efficiently inhibit VEGF-induced migration of microvascular ECs and exhibited anti-angiogenic and anti-tumor activity in several mouse and human xenograft models [201]. In humans, ABT-510 alone or in combination with cytotoxic agents was tested in several Phase I clinical trials to treat a variety of advanced solid and soft cancers [202,203,204,205,206,207]. Overall, ABT-510 administration was safe and well-tolerated, with modest adverse effects with injection-site reactions and fatigue as the most frequent. The anti-tumor effect of ABT-510 varied depending on the cancer type. In principal, treatment with ABT-510 alone had a modest efficiency. However, combinational therapy with cytotoxic agents improved the efficiency of anti-cancer therapy. In Phase II trials [208,209,210], ABT-510 monotherapy led to stabilization of tumor growth and inhibition of tumor expression of proangiogenic factors. In overall, treatment with ABT-510 alone showed a moderate efficiency in Phase II clinical studies by providing only a modest prolongation of overall survival of patients. Thus, based on the results of Phase II trials, clinical application of ABT-510 in combination with other anti-cancer agents was recommended.

In the context of cardiovascular therapy, anti-angiogenic approaches tested in tumors may be helpful in graft atherosclerosis. Mapping of salutary and deleterious effects to different TSP domains will provide an option to construct other TSP-targeting agents for widespread cardiovascular pathology such as MI and heart failure. In heart hypertrophy, atherosclerosis, heart failure, and MI, the down-regulation of the TSP/CD47 axis to enhance angiogenesis and restore NO-dependent signaling would be beneficial [121]. CD47 blockade with a monoclonal antibody was preclinically tested in animal models of ischemia resulted in improvement of angiogenesis and great increases in tissue survival [109,211].

So far, assessment of therapeutic effects CD47 blockade with a monoclonal antibody undergoes transition from the preclinical phase to clinical evaluation. The main purpose of these Phase I clinical trials is to check biosafety/tolerability of a CD47 antibody CC-90002 and find an optimal dose for treatment of advanced hematological neoplasms in combination with Rituximab, an anti-CD20 monoclonal antibody (trial NCT02367196) or for monotherapy of acute myeloid leukemia and high-risk myelodysplastic syndrome (trial NCT02641002). Another humanized anti-CD47 monoclonal antibody, Hu5F9-G4, will be clinically tested alone for treatment of recurrent/refractory acute myeloid leukemia (trial NCT02678338) and advanced solid malignancy or lymphoma (trial NCT02216409) [212]. At present, patients are enrolled for these clinical studies. Expected beneficial effects of this immunotherapy involve the inhibition of tumor angiogenesis and invasion, decrease of tumor-induced macrophage apoptosis and functional impairment, and depletion of CD47-expressing cancer stem cells that are key contributors to tumor relapse and chemoresistance [213]. In a case of the evident tolerability to antibodies, these trials will proceed to the Phase II.

In order to target a profibrotic activity of TSP1 through the activation of TGF-β, the activation sequence (LKSL) in the TSP1 molecule essential for the interaction with the latency-associated peptide (LAP) was mapped [214] and an LKSL peptide was developed [215]. The peptide antagonizes TSH1 binding to LAP and inhibits TGF-β liberation from the latent complex with LAT. The anti-fibrotic activity of the LKSL peptide was demonstrated in various animal models including experimental models of liver fibrosis [216], unilateral ureteral obstruction [217], diabetic nephropathy [218], and post-hemorrhagic hydrocephalus [219]. Regarding cardiovascular pathology, cardioprotective effects of LKSL peptide-mediated inhibition of TGF-β activation were observed in TAC-induced cardiomyopathy in type 1 diabetic rats. Diabetic rats treated with the LKSL peptide did not develop cardiac fibrosis and had improved heart function [52]. However, in a recent study, detrimental effects of implication of this peptide to treat angiotensin II-induced abdominal aortic aneurysm in ApoE-deficient mice were observed [220]. LKSL-dependent suppression of TGF-β activation further aggravated abdominal aortic aneurysm associated with increase of aortic diameter, adverse atherosclerosis within the aortic arch, and aortic elastin fragmentation due to down-regulation of the TGF-β-target gene lysyl oxidase-like 1 (LOXL1), an enzyme involved in cross-linking of elastin [221]. These data suggest for protective role of TGF-β against aortic aneurism. Inhibition of TGF-β signaling may therefore have deleterious consequences by impairing vascular ECM repair and promoting aortic infiltration of inflammatory cells. However, in overall, LKSL-mediated suppression of TSH1-dependent TGF-β activation showed beneficial results in inhibition of tissue fibrosis and should be further explored to prevent or diminish the advanced profibrotic response in hypertrophic hearts or in post-MI cardiac repair.

Since the regenerative potential of human heart is limited, MI-induced loss of cardiomyocytes can result in heart failure and death. Stem cell therapy has emerged as a promising strategy for healing cardiac injury, directly or indirectly, and seems to offer functional benefits to patients. Cardiac stem cell therapy involves using of hematopoietic, mesenchymal, and cardiac stem cells for regenerative purposes. However, a common challenge is to increase the retention and survival of engrafted cells at the injured site in order to strengthen their chances for proliferation and differentiation to functional cardiomyocytes [222]. To enhance the regenerative and prosurvival capacity, stem cells are subjected to ischemic/pharmacological preconditioning before transplantation. For example, regenerative properties of CD34^+^ hematopoietic progenitor cells from diabetic patients with atherosclerosis are frequently reduced and impaired. Treatment of CD34^+^ progenitors with TSP1-derived peptide RFYVVMWK promotes expression of TSP-1, integrins, and P-selectin that in turn increases adhesion capability and retention of the autologous progenitor cell engraft although do not affect apoptosis and viability [223]. Hypoxic exposure of adipose tissue-derived mesenchymal cells from aged mice improve their functionality by decreasing expression of anti-angiogenic, prothrombotic, and profibrotic molecules such as TSP-1, plasminogen activator inhibitor-1 (PAI-1), and TGF-β [224]. Preconditioning of mesenchymal stem cells with oxytocin significantly stimulates their therapeutic potential, angiogenic properties, and resistance to hypoxia and apoptosis through induction of various prosurvival and anti-apoptotic factors including TSP-1 [225].

## 12. Conclusions

**Figure 2:**
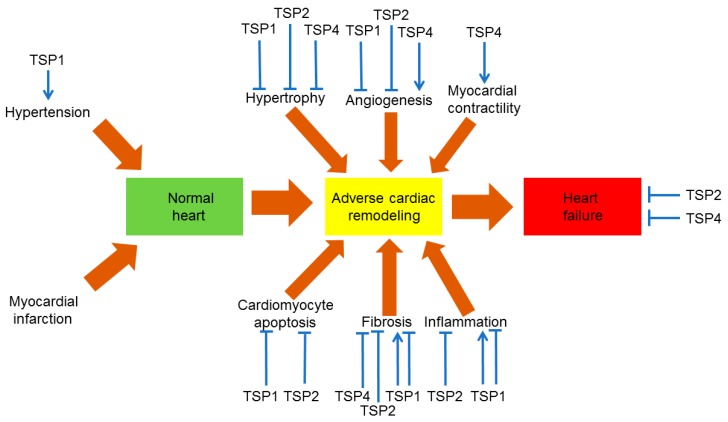
The role of thrombospondins (TSPs) in cardiovascular pathology. TSP1, TSP2, and TSP4 are the best preclinically studied TSPs in experimental models of cardiovascular pathology involving knockout or overexpression of these TSPs. Sharp arrows define stimulatory effects. Other type of arrows defines inhibitory effects.

Effects of TSPs on the mechanisms of cardiac remodeling are represented in Figure 2. Table 1 recapitulates TSP-dependent actions in the cardiovascular system. In summary, TSP1, TSP2, and TSP4 possess protective properties against heart hypertrophy since their deletion in animal models of pressure overload results in aberrant remodeling [49,50,59,144,147]. Lack of TSP1 and TSP2 activates MMP production and causes dilation of the left ventricle whereas altered TSP1-dependent TGF-β activation disturbs conversion of fibroblasts to myofibroblasts and down-regulates cardiac matrix synthesis [144]. In a murine pressure overload model, loss of TSP4 resulted in increased cardiac mass and fibrosis [50]. TSP4 also protects from abnormal heart remodeling through induction of stretch-mediated enhancement of myocardial contraction in pressure overload and prevention of the ER stress in cardiomyocytes [49,63]. On the basis of the role in heart hypertrophy, TSP1 and TSP2 would be suggested to influence post-MI remodeling but their effects are needed to be evaluated.

In failing hearts, TSP2 and TSP4 protect cardiac ECM from adverse remodeling. In TSP-deficient ageing heart, systolic function is altered while fibrinogenesis and cardiac dilatation are activated [54]. Also, an advanced cardiac muscle cell death, inflammation, MMP-2 activation and aberrant collagen cross-linking was observed [54]. TSP2 possess cardioprotective properties against heart failure in viral myocarditis by repressing inflammation, fibrotic response, and cardiomyocyte death [157]. In doxorubicin-induced cardiomyopathy, the anti-hypertrophic activity of TSP2 is related to the inhibition of apoptosis of cardiac muscle cells and preserving ECM from the damage [51]. As in a case of TSP2, TSP4 deletion results in fibrosis, altered diastolic function, and depressed systolic function denoting the involvement of TSP4 in the regulation of ECM composition [50]. Finally, TSP4 appears to possess the proatherosclerotic activity since ApoE-deficient mice lacking TSP4 had reduced macrophage accumulation in the plaques due to reduced proinflammatory activation of ECs and decreased recruitment of inflammatory leukocytes to the endothelium [31].

So far, the most comprehensive functional assessment of the physiological and pathological roles was done only for TSP1, a founder member of the TSP family. Less complete results were obtained for TSP2 and TSP4. From the scientific literature, there is a profound data deficit about the function of TSP3 and TSP5. Although TSP3 is expressed by VSMCs at significant levels, its expression in the homeostatic myocardium seems to be absent [42]. However, stimulation of cardiac fibroblasts with a peptide matricryptin generated by a limited proteolysis of collagen Iα1 by MMP-2/9 results in the induction of TSP3 expression. Along with TSP3, matricryptin induces production of many ECM structural and regulatory proteins that contribute to post-MI cardiac repair and promote scar formation and angiogenesis [226]. This effect of matricryptin has a therapeutic promise and therefore should be further evaluated. Since TSP1 and TSP2 inhibit proteolytic activation of MMP-2 and MMP-9 [227], these TSPs can potentially suppress cardiac expression of TSP3. Therefore, assessing reciprocal TSP regulation would be intriguing. Thus, future prospects in the thrombospondin-related research may ultimately concern investigation of TSP3 and TSP5 functions. Further, TSPs have a variety of binding partners and the number of TSP ligands is growing. However, regulatory mechanisms of binding these ligands are widely unknown. Actually, ligand binding is spatially and temporally regulated, and it would be of great interest to reveal these regulatory patterns and recognize their functional significance.

Overall, studies involving knockout mice indicate that deficiency of TSP1, TSP2, and TSP4 appears to be deleterious in cardiovascular pathology. Therefore, enhancing of cardiac TSP-dependent signaling in stressful settings such as pressure overload may be profitable. Precise analysis of the relationship between the TSP structure and function, identification of new receptors, and functional mapping of various domains will be useful for the development of novel drugs to target TSPs and promote the gain in TSP-dependent signaling pathways.

## Figures and Tables

**Table 1:** The role of thrombospondins in cardiovascular physiology and pathology

Characteristics	TSP1	TSP2	TSP3	TSP4	TSP5
Expression in the vascular wall	Yes	Yes	Yes	Yes	Yes
Expression in the atherosclerotic plaque	Yes	Yes	Yes	Yes	Yes
Cardiac expression	Yes	Yes	No?	Yes	Unknown
Angiogenesis in the myocardium	Inhibition	Inhibition	Unknown	Activation	Unknown
Up-regulated expression in cardiac remodeling	Yes	Yes	Yes	Yes	Yes
Inhibition of MMP-2/3/9	Yes	Yes	No	No	No
Cardiac fibrosis	Activation/Inhibition	Inhibition	Unknown	Inhibition	Unknown
VSMC proliferation/hyperplasia	Activation	Activation	Unknown	No effect	Inhibition
Blood pressure	Vasoconstriction	Vasoconstriction	Unknown	Unknown	Unknown
Inflammation	Activation/Inhibition	Inhibition	Unknown	Activation (moderate)	Unknown
Effects on macrophages	Stimulation of phagocytosis Foam cell formation	Unknown	Unknown	Recruitment to the plaque	Unknown
Plaque progression	Activation	Unknown	Unknown	Activation	Unknown
Oxidative stress	Activation	Unknown	Unknown	Unknown	Unknown
Cardiomyocyte apoptosis	Inhibition	Inhibition	Unknown	Unknown	Unknown
Cardiac contractility	No effect	Unknown	Unknown	Activation	Unknown
Cardiac hypertrophy	Inhibition	Inhibition	Unknown	Inhibition	Unknown
Heart failure	Inhibition?	Inhibition	Unknown	Inhibition	Unknown

Abbreviations: MMP, matrix metalloproteinase; TSP, thrombospondin; VSMC, vascular smooth muscle cell.
